# Pathomechanisms of Dermatologic Adverse Effects of Targeted Therapies in Pediatric Solid Tumors: A Narrative Review

**DOI:** 10.1007/s11912-026-01854-8

**Published:** 2026-11-05

**Authors:** Laura Klaus, Edit Brückner, Péter B. Botos, Márta Medvecz, Péter Holló, Attila J. Szabó, Sarolta Kárpáti, Miklós Garami

**Affiliations:** 1 Pediatric Center, Semmelweis University, Budapest, H-1094 Hungary; 2 Department of Dermatology, Venereology and Dermatooncology, Semmelweis University, Budapest, H-1085 Hungary

**Keywords:** Pediatric oncology, Targeted therapy, Dermatologic toxicity, MEK inhibitors, BRAF inhibitors, EGFR inhibitors

## Abstract

**Purpose of Review:**

Targeted anticancer therapies have transformed the treatment of pediatric solid tumors but are associated with a distinct spectrum of dermatologic adverse effects that may impair quality of life and compromise treatment adherence. This review summarizes the molecular and cellular mechanisms underlying these toxicities and integrates current mechanistic evidence with their clinical manifestations, prevention, and management.

**Recent Findings:**

Recent mechanistic and clinical evidence indicates that targeted therapy–related cutaneous toxicities arise from pathway-specific disruption of epidermal, vascular, and immune homeostasis. Major mechanisms include paradoxical MAPK activation during BRAF inhibition; impaired keratinocyte proliferation, differentiation, and barrier function during MEK, EGFR, and mTOR inhibition; endothelial and epidermal injury associated with VEGFR-targeting multikinase inhibitors; and loss of immune tolerance during immune checkpoint blockade. These mechanisms contribute to characteristic phenotypes including papulopustular eruptions, xerosis, paronychia, hand–foot skin reaction, pigmentary alterations, and immune-mediated dermatoses. Pediatric-specific mechanistic and prospective dermatologic data remain limited, and evidence for several newer targeted agents is largely extrapolated from adult studies.

**Summary:**

Dermatologic toxicities of targeted therapies reflect predictable perturbations of signaling pathways that are also essential for normal skin homeostasis. Understanding these pathomechanisms may facilitate early recognition, mechanism-based prevention and treatment, and preservation of anticancer therapy. Pediatric-specific prospective studies and standardized dermatologic outcome reporting are needed.

## Introduction

Over the past two decades, the rapid expansion of molecularly targeted therapies has transformed the treatment landscape of pediatric oncology. By selectively inhibiting oncogenic signaling pathways, these agents have improved tumor control and, in some settings, reduced systemic toxicities compared with conventional chemotherapy [1]. Their integration into multimodal regimens for pediatric solid tumors—such as gliomas, neuroblastomas, and sarcomas—has significantly advanced precision medicine in children, enabling individualized treatment strategies based on tumor-specific molecular alterations.

Despite these advances, dermatologic adverse effects are common during targeted therapy and, although rarely life-threatening, can substantially affect quality of life and treatment adherence [2]. These cutaneous toxicities differ from those seen with cytotoxic chemotherapy and are thought to result from on-target or paradoxical effects on epidermal signaling pathways and immune regulation. In pediatric patients, whose skin physiology and repair capacity differ from adults, such adverse events present unique diagnostic and management challenges [3].

This review examines the molecular and cellular basis of dermatologic toxicities associated with targeted therapies in pediatric solid tumors. Mechanistic and clinical findings are considered together to relate pathway inhibition to the resulting cutaneous reactions. The scope is limited to peer-reviewed studies with defined pediatric populations or mechanistic relevance, emphasizing findings that inform prevention, early recognition, and management of treatment-limiting dermatologic events.

## Literature Review Approach

This narrative review summarizes current evidence regarding the molecular mechanisms, clinical manifestations, diagnosis, and management of dermatologic adverse effects associated with targeted therapies used in pediatric solid tumors.

Relevant literature published between 2000 and 2026 was identified through searches of PubMed/MEDLINE, Embase, Web of Science, Scopus and other major biomedical databases. Additional articles were identified through manual review of reference lists from key publications. Priority was given to peer-reviewed pediatric studies, clinical trials, translational investigations, consensus guidelines, and review articles that provided mechanistic or clinically relevant information regarding dermatologic toxicities associated with targeted anticancer therapies. Where full peer-reviewed publications were unavailable for recently introduced pediatric therapies, relevant conference abstracts reporting pivotal or updated clinical trial data were also considered.

Because the objective of this review was to provide a focused mechanistic and clinical overview rather than a systematic evidence synthesis, formal systematic review methodology, quantitative meta-analysis, and risk-of-bias assessment were not performed. The selected literature was interpreted narratively, with emphasis on biologic plausibility, consistency across studies, and relevance to pediatric oncology practice.

## Results

### Current Evidence

The literature reviewed for this narrative synthesis included clinical trials, cohort studies, mechanistic investigations, review articles, and pediatric case series addressing dermatologic toxicities associated with targeted therapies used in pediatric oncology. The strength of available evidence varied across therapeutic classes, with the most robust data available for BRAF/MEK-targeted therapies in pediatric low-grade glioma and MEK inhibitors in neurofibromatosis type 1–associated plexiform neurofibromas. Although reporting of dermatologic outcomes was heterogeneous, the underlying mechanistic findings were generally consistent across studies [2–6]. The principal pediatric targeted therapies, their clinical indications, dermatologic adverse effects, and underlying molecular mechanisms are summarized in Table 1.

**Table 1:** Pediatric targeted therapies: clinical evidence, dermatologic toxicities and underlying pathomechanisms

Therapy/Class	Pediatric Status/Indication	Highest Level of Evidence	Main Clinical Effect	Key Dermatologic Adverse Effects	Proposed Pathomechanism	Dermatologic Management*
Dabrafenib + Trametinib	BRAF V600E-mutant pLGG (≥ 1 year)	Randomized phase II clinical trial; FDA/EMA-approved [5]	Improved objective response rate and progression-free survival	Rash, xerosis, acneiform dermatitis, paronychia; markedly reduced KA/cuSCC incidence vs. BRAF inhibitor monotherapy	Paradoxical RAF dimerization activates MAPK/ERK signaling in wild-type keratinocytes during BRAF inhibition; concomitant MEK inhibition suppresses this pathway, reducing keratinocyte hyperproliferation and secondary epithelial neoplasia.	Gentle skin care, emollients, and photoprotection; eruption-specific topical anti-inflammatory treatment; dermatologic evaluation for persistent or severe reactions [7]
Selumetinib	NF1-associated symptomatic, inoperable plexiform neurofibroma; recurrent/progressive pLGG	Phase II trial; FDA/EMA-approved [8]	Tumor shrinkage, functional improvement, improved quality of life	Acneiform eruption, paronychia, xerosis, hair depigmentation and texture changes	Reduced ERK signaling stabilizes KLF4, promoting IL-36γ and IL-8 expression, neutrophilic inflammation, impaired keratinocyte proliferation, and altered epidermal differentiation.	Preventive skin care and emollients; topical corticosteroids or antimicrobial treatment according to lesion type and anatomical site; age-appropriate systemic therapy for selected severe acneiform eruptions; periungual [9] care
Mirdametinib	NF1-associated symptomatic plexiform neurofibroma (≥ 2 years)	Pivotal phase IIb (ReNeu); FDA-approved [10]	Durable tumor volume reduction and improved quality of life	Acneiform dermatitis, xerosis, paronychia, rash	As a MEK inhibitor, mirdametinib disrupts ERK-dependent keratinocyte homeostasis; inflammatory mechanisms involving IL-36γ and IL-8 are inferred from mechanistic studies of MEK inhibition rather than demonstrated specifically for mirdametinib	Age- and pubertal-stage–adapted skin care; emollients and photoprotection; lesion-specific topical treatment; specialist-guided systemic treatment for selected moderate-to-severe reactions [11]
Everolimus	TSC-associated SEGA	Phase III (EXIST-1); FDA/EMA approved [12]	Significant reduction in SEGA volume	Stomatitis, rash, xerosis, acneiform eruption	mTORC1 inhibition reduces STAT3 activation, impairing keratinocyte survival, epidermal regeneration, and barrier maintenance while increasing susceptibility to apoptosis.	Oral hygiene, bland alcohol-free mouth rinses, and avoidance of mucosal irritants; topical corticosteroids for selected stomatitis lesions when appropriate; emollients and symptom-directed topical treatment for cutaneous eruptions [13]
PD-1/PD-L1 inhibitors	Pediatric melanoma and selected pediatric indications	Phase I/II pediatric clinical studies [14]	Durable responses in selected patients	Morbilliform, psoriasiform and lichenoid eruptions, vitiligo-like depigmentation, bullous pemphigoid, SJS/TEN	Loss of peripheral immune tolerance activates autoreactive T cells. IFN-γ–CXCL9/CXCL10 signaling recruits CXCR3-positive CD8 + lymphocytes, while IL-15 supports tissue-resident memory T cells. Severe reactions result from cytotoxic T-cell–mediated keratinocyte apoptosis.	Severity- and morphology-guided management; emollients and topical corticosteroids for mild inflammatory eruptions; prompt dermatologic assessment for severe or blistering reactions; systemic corticosteroids and immunotherapy interruption when indicated [15]
VEGFR / Multikinase inhibitors	Primarily off-label pediatric solid tumors	Phase I/II studies and pediatric cohort studies	Disease stabilization and tumor control	Hand-foot skin reaction, xerosis, paronychia, fissures	Impaired angiogenesis and endothelial repair; mechanical stress–induced endothelial injury; sorafenib-specific s-HBEGF/EGFR–SIRT1 and OAT6/MAP3K7 signaling	Emollients and reduction of pressure and friction; topical urea-based keratolytics and corticosteroids for selected hand–foot skin reactions; dermatologic assessment and oncologist-guided treatment modification for severe toxicity [16, 17].
ALK inhibitors	ALK-positive inflammatory myofibroblastic tumor and other ALK-driven malignancies	Phase I/II studies [18]	High antitumor activity	Photosensitive dermatitis, lichenoid eruptions	Mechanism of photosensitivity remains incompletely defined; lichenoid reactions are consistent with delayed T-cell–mediated keratinocyte injury	Strict photoprotection with broad-spectrum SPF 50 or higher sunscreen and protective clothing; topical corticosteroids for selected lichenoid eruptions; dermatologic assessment and oncologist-guided treatment modification for severe reactions [19, 20]
NTRK inhibitors	Tumor-agnostic NTRK fusion-positive pediatric tumors	Pooled phase I/II pediatric studies [21]	High objective response rate and durable responses	Rash, pruritus; other cutaneous reactions are incompletely characterized.	Reduced NGF–TRKA signaling may theoretically affect keratinocyte homeostasis, epidermal regeneration, and skin barrier integrity; however, a direct causal link to NTRK inhibitor–associated cutaneous toxicity has not been established.	Gentle skin care and emollients; symptom-directed treatment of pruritus and inflammatory eruptions; dermatologic assessment for persistent or severe reactions, with oncologist-guided treatment modification when indicated [22]
RET inhibitors	RET-altered thyroid carcinoma and RET fusion-positive tumors (≥ 2 years)	Phase I/II pediatric trial data (LIBRETTO-121; conference report); FDA-approved [23]	Durable disease control	Xerosis, eczematous dermatitis, pruritus, mucositis	Likely impairment of epithelial homeostasis and barrier maintenance with possible immune-mediated inflammatory mechanisms; precise molecular pathway remains incompletely defined.	Topical corticosteroids for localized inflammatory eruptions; oral antihistamines for associated pruritus; supportive oral care for stomatitis; specialist-guided systemic treatment and oncologist-directed treatment interruption for severe reactions [24]
Dinutuximab / Dinutuximab beta	High-risk neuroblastoma following multimodal therapy	Randomized phase III registrational trial; FDA/EMA-approved [25]	Improved event-free and overall survival	Erythema, urticaria, pruritus, rash, inflammatory flares	Binding to GD2 induces complement-dependent cytotoxicity and antibody-dependent cellular cytotoxicity, with cytokine release and Fcγ receptor-mediated immune activation producing inflammatory cutaneous reactions.	Antihistamine premedication and close monitoring during infusion; symptomatic management and temporary infusion interruption or rate reduction for mild cutaneous reactions; immediate infusion interruption and emergency management for severe hypersensitivity, with permanent discontinuation for life-threatening anaphylaxis [26, 27]

*Dermatologic management recommendations are based on available clinical studies, expert guidance, and drug-specific prescribing information. Several recommendations, particularly for ALK, NTRK, and RET inhibitors, are extrapolated from adult populations and have not been validated as pediatric-specific management protocols

### Pathomechanisms by Therapeutic Target

For readability, the mechanisms are presented by therapeutic class and grouped according to the principal signaling pathway or cellular process targeted: inhibitors of MAPK and mTOR signaling, other tyrosine kinase inhibitors, cell-cycle and epigenetic modulators, and antibody-based immunotherapy.

### Inhibitors of MAPK and mTOR Signaling

#### BRAF Inhibitors

**Fig. 1:**
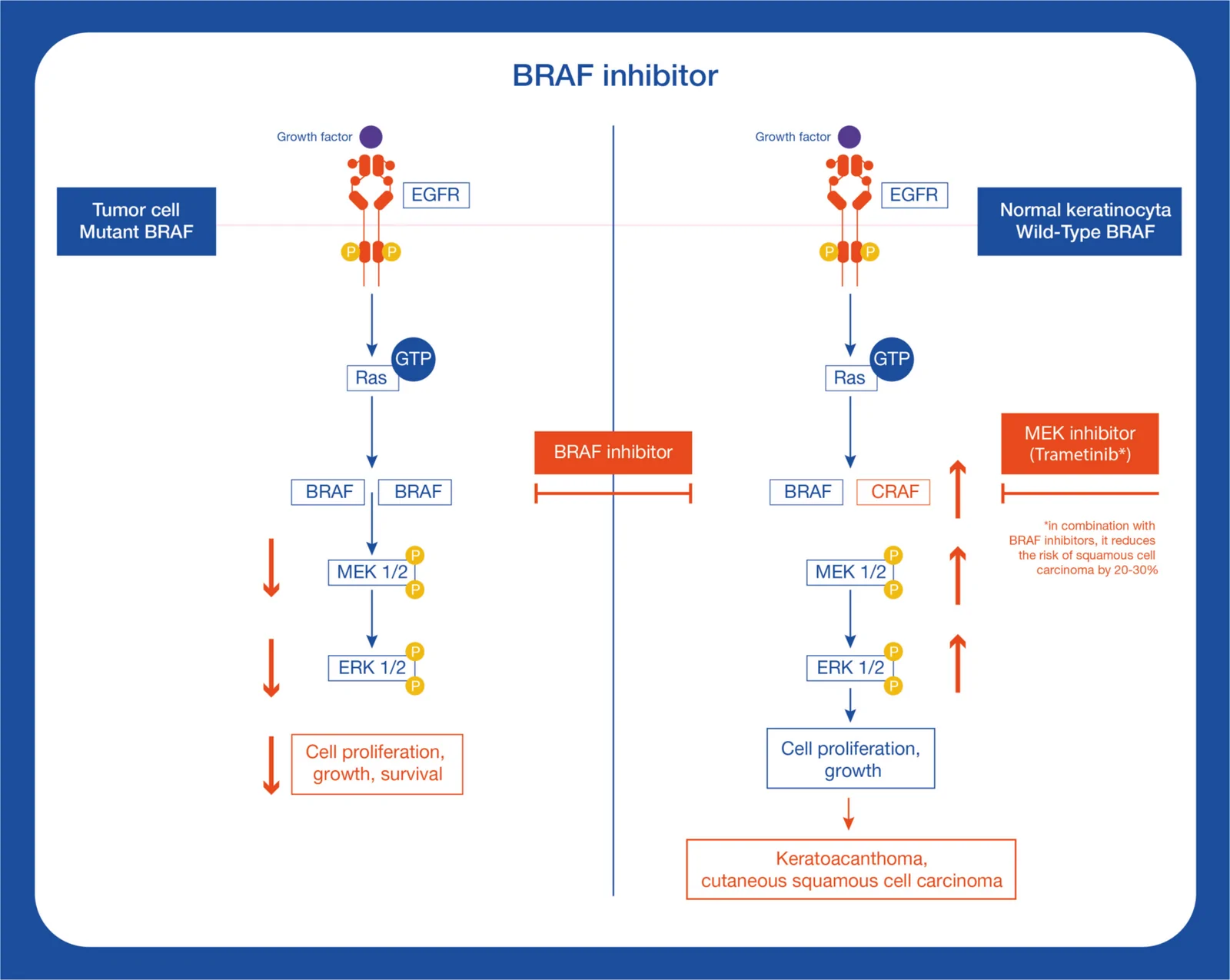
Molecular Mechanisms of BRAF Inhibitor-Induced Cutaneous. Toxicity BRAF inhibitors selectively suppress constitutively active mutant BRAF signaling in tumor cells but can paradoxically activate MAPK signaling in keratinocytes with wild-type BRAF. In the presence of upstream RAS activity, ATP-competitive BRAF inhibitors promote RAF dimerization and transactivation of CRAF, leading to MEK and ERK phosphorylation. Sustained ERK activation promotes keratinocyte hyperproliferation and aberrant differentiation, contributing to hyperkeratotic lesions, keratoacanthomas, and cutaneous squamous cell carcinomas. Concomitant MEK inhibition suppresses downstream ERK activation and substantially reduces the incidence of BRAF inhibitor-associated keratoacanthomas and cutaneous squamous cell carcinomas

BRAF inhibitors (dabrafenib, vemurafenib, and encorafenib) selectively inhibit constitutively active BRAF V600 mutant kinases in tumor cells but paradoxically activate MAPK signaling in wild-type keratinocytes. In the presence of upstream RAS activation, ATP-competitive BRAF inhibitors promote RAF dimerization and transactivation of the uninhibited RAF protomer, most commonly CRAF, resulting in sustained MEK and ERK phosphorylation despite pharmacologic BRAF inhibition. Persistent ERK activation drives keratinocyte proliferation, accelerates cell-cycle progression, and promotes epidermal hyperplasia, ultimately leading to hyperkeratotic lesions, verrucous keratoses, keratoacanthomas, and cutaneous squamous cell carcinomas. Activating HRAS mutations are frequent in BRAF inhibitor–associated keratoacanthomas and cutaneous squamous cell carcinomas, indicating preferential expansion of pre-existing RAS-mutant keratinocyte clones rather than de novo malignant transformation. Ultraviolet radiation may further increase susceptibility by generating or selecting keratinocyte clones harboring oncogenic RAS mutations. In clinical practice, combined BRAF/MEK inhibition (trametinib, cobimetinib, or binimetinib) suppresses paradoxical ERK activation downstream of RAF dimers, thereby preventing expansion of RAS-primed keratinocyte clones while simultaneously enhancing antitumor MAPK pathway inhibition. Consequently, combination therapy is associated with a substantially lower incidence of keratoacanthomas and cutaneous squamous cell carcinomas than BRAF inhibitor monotherapy [28–33] (Fig. 1).

#### MEK and EGFR Inhibitors

**Fig. 2:**
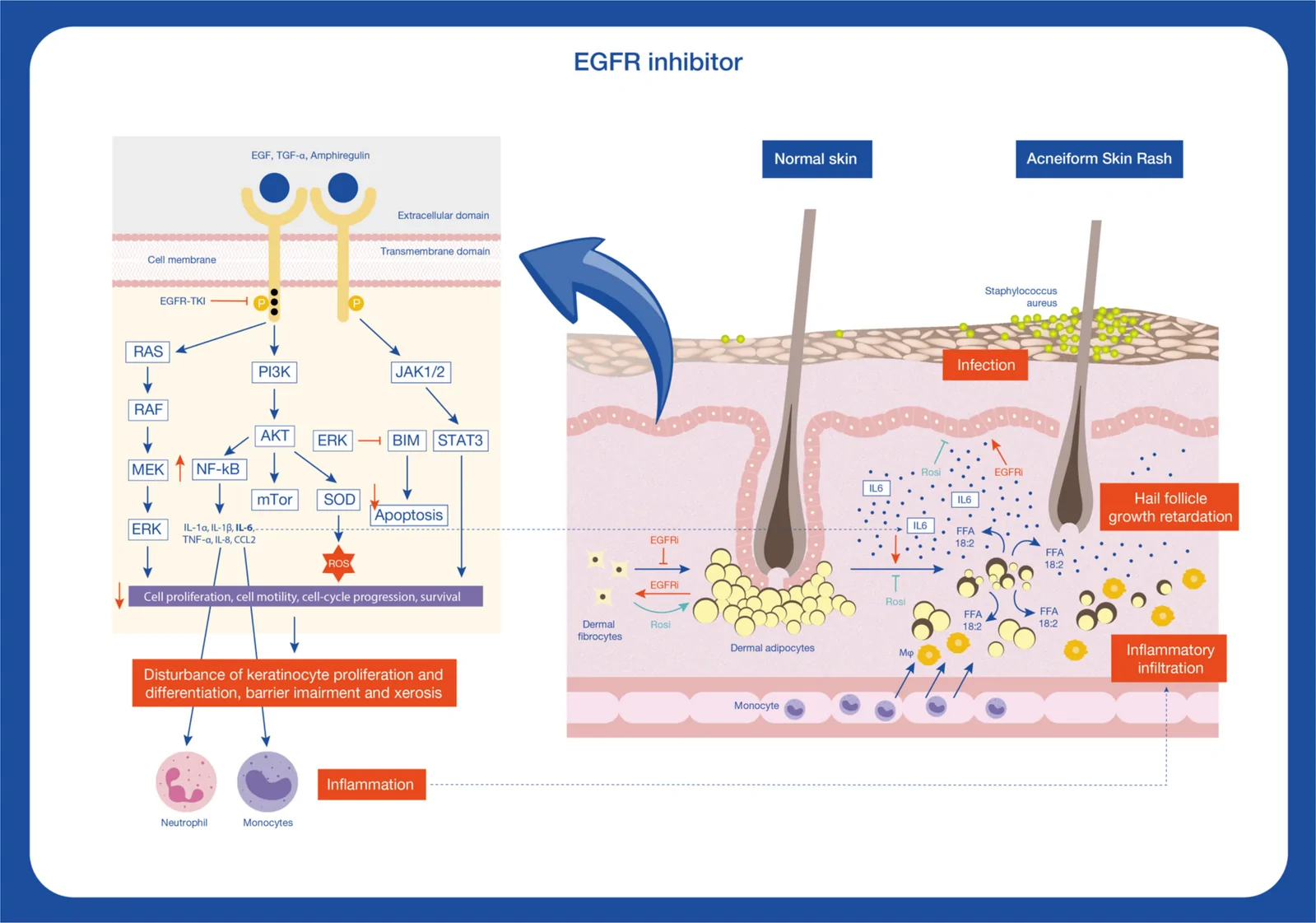
Pathomechanisms of EGFR Inhibitor–Induced Cutaneous. Toxicity EGFR inhibition suppresses MAPK/ERK and PI3K/AKT signaling in keratinocytes, impairing proliferation, differentiation, and epidermal barrier integrity. Loss of EGFR-mediated homeostatic signaling promotes activation of NF-κB and AP-1, resulting in increased production of proinflammatory cytokines, including IL-1β, IL-6, IL-8, and TNF-α. Cytokine release contributes to recruitment of neutrophils and macrophages and amplifies local inflammation. In parallel, altered dermal adipocyte function and reduced antimicrobial defense may facilitate bacterial colonization and further exacerbate inflammation. Hair follicle injury and follicular inflammation ultimately lead to papulopustular (acneiform) eruptions characteristic of EGFR-targeted therapy

**Fig. 3:**
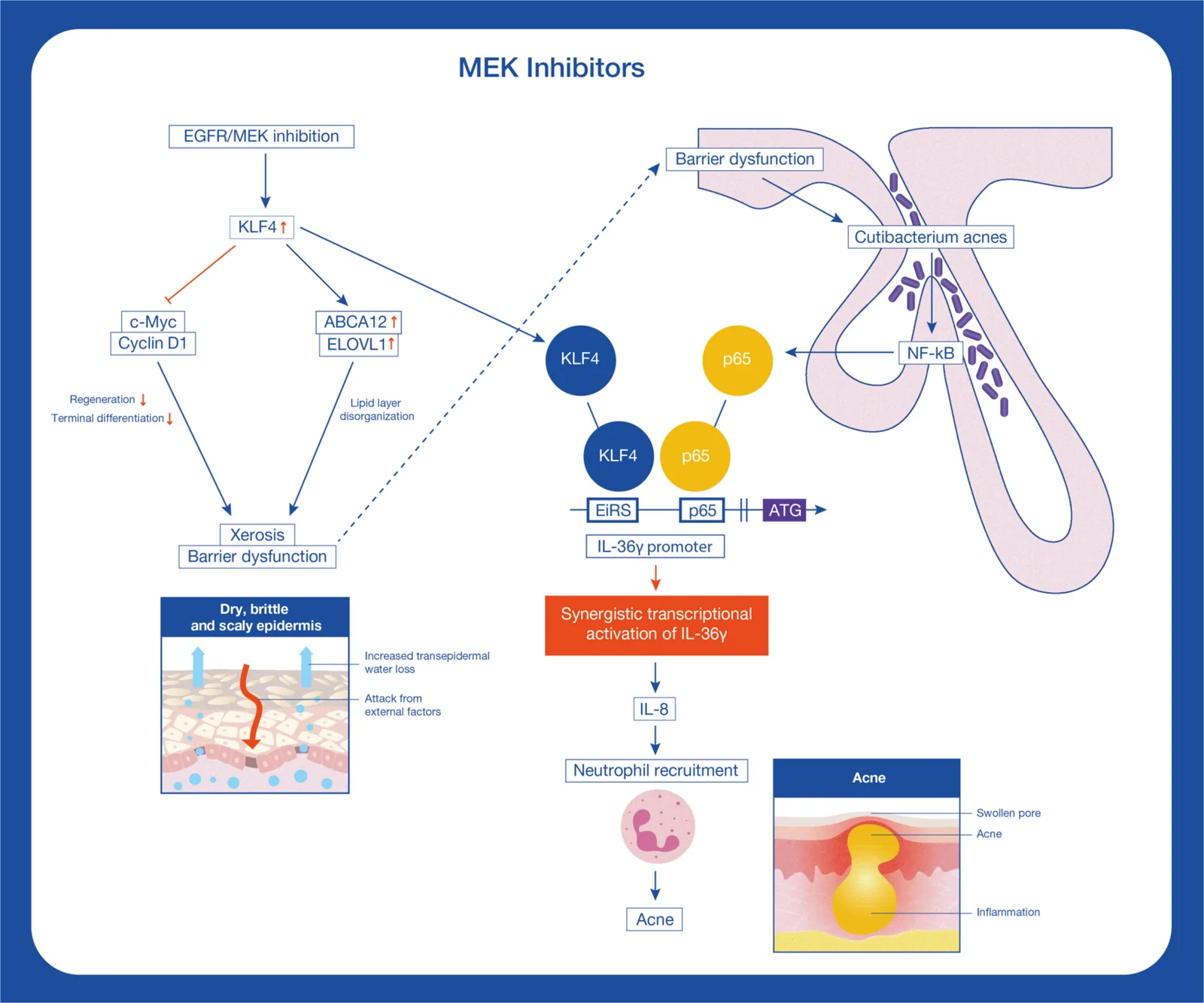
Mechanisms of MEK Inhibitor–Induced Acneiform Skin Toxicity. EGFR and MEK inhibitors suppress MAPK signaling (EGFR–RAS–RAF–MEK–ERK) in keratinocytes, leading to reduced ERK phosphorylation and consequent nuclear accumulation of KLF4. KLF4 enhances IL-36γ transcription and also regulates lipid metabolism genes (e.g., ABCA12, ELOVL1), contributing to barrier integrity, while simultaneously repressing proliferation-associated genes (c-Myc, Cyclin D1), thereby promoting keratinocyte differentiation. In the presence of Cutibacterium acnes, bacterial components activate TLR2 and NOD1/NOD2 receptors, triggering MyD88-dependent NF-κB and MAPK signaling and increasing the production of IL-36γ, IL-1β, IL-6, and tumor necrosis factor (TNF)-α. KLF4-driven and NF-κB–mediated signals synergistically amplify IL-36γ expression, which induces IL-8 production, leading to neutrophil recruitment and acneiform inflammation

MEK inhibitors (selumetinib, trametinib, and cobimetinib) directly suppress MAPK signaling downstream of RAF, whereas EGFR inhibitors (erlotinib and panitumumab) inhibit upstream EGFR signaling, resulting in reduced ERK phosphorylation and increased nuclear accumulation of Kruppel-like factor 4 (KLF4) in epidermal keratinocytes. KLF4 enhances IL-36γ transcription while suppressing proliferation-associated genes, including c-MYC and CCND1, and promotes terminal keratinocyte differentiation. In the presence of *Cutibacterium acnes*, activation of TLR2- and NOD-dependent innate immune signaling synergizes with KLF4-mediated transcriptional regulation to amplify IL-36γ and IL-8 production through NF-κB–dependent pathways. The resulting neutrophil-dominant inflammation contributes to the characteristic acneiform eruptions seen with MEK and EGFR inhibitors [34–41] (The molecular mechanisms underlying EGFR inhibitor-induced cutaneous toxicity are summarized in Fig. 2 ). MEK inhibition also activates an IRF1–IFN-κ–STAT1 signaling axis in keratinocytes, increasing CCL2 and CXCL10 expression and inducing a type I interferon–associated response that may further contribute to cutaneous inflammation [42]. This mechanism also explains the characteristic folliculocentric distribution of EGFR and MEK inhibitor–induced eruptions, as Cutibacterium acnes preferentially colonizes pilosebaceous units where TLR2-dependent signaling amplifies IL-36γ production [43]. Histopathology typically shows suppurative folliculitis with neutrophil-predominant perifollicular and periadnexal infiltrates, often accompanied by apoptotic keratinocytes, consistent with inflammation centered on follicular keratinocyte injury [44]. (Fig. 3) In pediatric patients, acneiform eruptions appear more frequently in older children and adolescents than in younger children, suggesting that age-dependent differences in sebaceous gland activity and follicular biology may further modulate susceptibility to MEK inhibitor–associated follicular inflammation [45]. MEK inhibition also disrupts keratinocyte proliferation and migration, impairing epidermal barrier integrity and promoting the release of pro-inflammatory cytokines and chemokines. These changes may contribute to xerosis, eczematous dermatitis, and susceptibility to secondary cutaneous infections [44].

#### mTOR Inhibitors

mTOR inhibitors (everolimus and temsirolimus) impair epidermal homeostasis primarily through inhibition of mTORC1 signaling, resulting in reduced protein synthesis, diminished keratinocyte proliferation, and impaired epidermal regeneration. Everolimus attenuates STAT3 signaling in keratinocytes and increases their susceptibility to apoptosis. As STAT3 is a key transcriptional regulator of several anti-apoptotic proteins, including Bcl-2, Mcl-1, and survivin, reduced STAT3 activity may further compromise keratinocyte survival, although the contribution of these downstream mediators to mTOR inhibitor–associated cutaneous toxicity remains incompletely defined. These alterations impair keratinocyte differentiation and epidermal barrier integrity. Reduced STAT3 activity may also favor pro-inflammatory signaling and contribute to maculopapular eruptions, xerosis, and oral mucositis during mTOR inhibitor therapy [46–49].

### Other Tyrosine Kinase Inhibitors

#### ALK Inhibitors

ALK inhibitors (crizotinib, alectinib, ceritinib, and lorlatinib) have been associated with phototoxic and delayed T-cell–mediated cutaneous reactions. The molecular basis of ALK inhibitor–associated photosensitivity has not been established, whereas lichenoid reactions are consistent with delayed T-cell–mediated keratinocyte injury. Direct mechanistic evidence remains limited, but these processes may account for the photosensitive and lichenoid reactions reported with ALK inhibitors [50–52].

#### VEGFR-Targeting Multikinase Inhibitors

VEGFR-targeting multikinase inhibitors (MKIs), including sorafenib, sunitinib, pazopanib, and regorafenib, induce hand–foot skin reaction (HFSR) through combined vascular and epidermal mechanisms. Inhibition of VEGFR signaling impairs endothelial cell survival, angiogenesis, and microvascular repair, resulting in reduced capillary integrity, diminished tissue perfusion, and delayed tissue regeneration. The palms and soles are particularly vulnerable because repetitive pressure and friction add mechanical microtrauma to the underlying microvascular injury. Microvascular injury increases vascular permeability and promotes the release of inflammatory mediators into the surrounding tissue, leading to secondary keratinocyte injury, altered epidermal differentiation, and impaired barrier homeostasis. In parallel, inhibition of additional kinase targets, including PDGFR, c-KIT, and TIE-2, may further impair dermal repair, extracellular matrix remodeling, and epidermal regeneration, thereby amplifying tissue susceptibility to mechanical injury. Together, endothelial dysfunction, mechanical stress, and keratinocyte injury produce the painful hyperkeratotic lesions characteristic of HFSR.

Recent mechanistic studies have identified additional molecular pathways that appear to contribute specifically to sorafenib-induced HFSR. Experimental evidence demonstrates that sorafenib suppresses the soluble heparin-binding epidermal growth factor (s-HBEGF)/EGFR signaling axis while simultaneously reducing the activity of the NAD⁺-dependent deacetylase SIRT1 through depletion of intracellular NAD⁺ pools. Loss of EGFR-mediated regenerative signaling together with impaired SIRT1-dependent cellular stress responses increases endothelial and keratinocyte vulnerability to mechanical injury, thereby amplifying local inflammation and tissue damage. Furthermore, nicotinamide supplementation restores intracellular NAD⁺ metabolism, reactivates SIRT1 signaling, and attenuates experimental HFSR, providing functional evidence for the involvement of this pathway. These molecular findings are specific to sorafenib, and whether they also contribute to HFSR caused by other VEGFR-targeting multikinase inhibitors is unknown.

Direct accumulation of selected multikinase inhibitors in epidermal keratinocytes may also contribute to HFSR independently of vascular injury. Organic anion transporter 6 (OAT6)-mediated intracellular uptake has been shown to facilitate keratinocyte toxicity through activation of MAP3K7-dependent cell death pathways, indicating that both endothelial dysfunction and direct epidermal toxicity participate in the pathogenesis of HFSR [53–56].

#### RET Inhibitors

RET inhibitors (selpercatinib and pralsetinib) have been associated with xerosis, pruritus, and eczematous dermatitis, but their cutaneous effects have not yet been mechanistically characterized. RET signaling contributes to epithelial homeostasis through regulation of cell survival, proliferation, and tissue repair. RET inhibition could impair epidermal regeneration and barrier maintenance, potentially predisposing to xerosis and secondary inflammation Disrupted epithelial homeostasis could also promote local inflammatory signaling and contribute to pruritus and eczematous dermatitis. These proposed mechanisms have not been demonstrated directly in human skin during RET inhibitor therapy [57–59].

#### NTRK Inhibitors

NTRK inhibitors (larotrectinib and entrectinib) block TRK signaling, including TRKA-mediated pathways expressed in human keratinocytes. In normal skin, NGF–TRKA signaling supports keratinocyte proliferation, migration, survival, epithelial repair, and epidermal differentiation. TRK inhibition could consequently interfere with epidermal repair and barrier function. Experimental data indicate that NGF–TRKA signaling regulates epidermal differentiation proteins, including filaggrin and involucrin, which may help explain the xerosis and eczematous reactions reported with TRK inhibition. Impaired NGF signaling has also been proposed to alter cutaneous neuroimmune interactions and promote pruritus, although direct evidence from patients receiving NTRK inhibitors is lacking. These pathways offer a possible explanation for the uncommon and generally nonspecific cutaneous events reported with TRK inhibitors, including rash, pruritus, xerosis, and dermatitis, but direct mechanistic evidence is sparse [60–64].

#### BCR-ABL/PDGFR Inhibitors

BCR-ABL/PDGFR inhibitors (imatinib and dasatinib) induce cutaneous adverse effects through combined inhibition of c-KIT, PDGFR, and ABL signaling pathways. In melanocytes, inhibition of c-KIT disrupts downstream MAPK/ERK and PI3K/AKT signaling, impairing melanocyte survival and melanogenesis and thereby contributing to hypo- and depigmentation. In parallel, inhibition of PDGFR signaling may impair dermal fibroblast function, extracellular matrix remodeling, and tissue repair, predisposing to xerosis and delayed epidermal regeneration. Epithelial stress may additionally promote release of alarmins such as IL-33, whereas IL-31 signaling has been implicated in pruritus through effects on sensory neurons and keratinocytes. The relevance of IL-31 and IL-33 signaling to BCR-ABL inhibitor–associated skin toxicity, however, is uncertain Among the proposed mechanisms, disruption of c-KIT–dependent melanocyte homeostasis and impaired dermal repair have the strongest supporting evidence [65–68]. Rare severe cutaneous adverse reactions, including Stevens–Johnson syndrome, are presumed to arise from HLA class I–restricted activation of cytotoxic CD8 + T cells, leading to keratinocyte apoptosis mediated by granulysin, perforin/granzyme B, and Fas–FasL signaling, although the initiating mechanism of imatinib-specific T-cell activation remains incompletely understood [69].

### Cell-Cycle and Epigenetic Modulators

#### CDK4/6 Inhibitors

Photosensitive dermatitis, dyschromia, and vitiligo-like depigmentation have been reported with CDK4/6 inhibitors (palbociclib, ribociclib, and abemaciclib), but mechanistic data are scarce. Experimental studies have established that SUMOylation plays an important role in keratinocyte differentiation and epidermal homeostasis One hypothesis is that CDK4/6 inhibition interferes with SUMO-dependent regulation of epidermal stress responses, increasing susceptibility to photosensitivity and pigmentary change Cell-cycle arrest in melanocytes has also been proposed as a possible basis for vitiligo-like depigmentation. Neither altered keratinocyte SUMOylation nor melanocyte dysfunction has yet been demonstrated directly during CDK4/6 inhibitor therapy [70–72].

#### HDAC Inhibitors

HDAC inhibitors (panobinostat) induce widespread histone and non-histone protein hyperacetylation, resulting in global epigenetic reprogramming of gene expression. These epigenetic alterations affect multiple cellular processes, including cell-cycle regulation, apoptosis, inflammatory signaling, and cellular stress responses. Experimental studies have shown that HDAC inhibition broadly alters inflammatory signaling, differentiation-related transcriptional programs, apoptosis, and cellular stress responses. However, direct mechanistic evidence linking these pathways specifically to panobinostat-induced cutaneous toxicity remains limited. Altered expression of cell-cycle regulators (e.g., p21), pro- and anti-apoptotic proteins (including BAX and BCL-2 family members), together with enhanced oxidative and endoplasmic reticulum stress responses, may further increase keratinocyte susceptibility to injury. These cellular effects could contribute to the erythematous, exfoliative, and acneiform reactions reported with panobinostat, although a direct mechanistic link to individual cutaneous phenotypes has not been established [73–76].

### Antibody-Based Immunotherapy

#### GD2-Targeting Immunotherapy

GD2-targeting immunotherapy with dinutuximab beta is a human/mouse chimeric IgG1 monoclonal antibody that exerts its antitumor activity primarily through complement-dependent cytotoxicity (CDC), antibody-dependent cellular cytotoxicity (ADCC), and antibody-dependent cellular phagocytosis (ADCP). Following binding to GD2-expressing neuroblastoma cells, activation of the classical complement cascade generates the anaphylatoxins C3a and C5a, while Fcγ receptor engagement activates natural killer cells, macrophages, and granulocytes, promoting tumor cell lysis and release of pro-inflammatory cytokines.

Because GD2 is physiologically expressed at low levels in peripheral nerve fibers and melanocytes, antibody binding may also induce on-target immune activation in normal tissues. Complement activation and cytokine release (including TNF-α, IL-1β, and IL-6) increase vascular permeability and promote mast-cell activation and histamine release, accounting for many of the pruritic, urticarial, erythematous, and infusion-related reactions seen during dinutuximab beta therapy. In contrast, current evidence suggests that these dermatologic toxicities result predominantly from secondary immune activation rather than direct keratinocyte injury.

Binding of dinutuximab beta to GD2 expressed on peripheral nerve fibers also contributes to treatment-related neuropathic pain through complement activation and local neuroinflammation. Rare pigmentary alterations may reflect on-target recognition of GD2-expressing melanocytes, although this association has not been mechanistically established [25, 26, 77–80].

#### Immune Checkpoint Inhibitors

Immune checkpoint inhibitors (ICIs) can induce cutaneous adverse events through disruption of immune regulatory mechanisms that normally maintain peripheral tissue tolerance. Experimental evidence indicates that programmed cell death protein 1 (PD-1) restrains the pathogenic activity of skin-infiltrating, antigen-specific CD8 + T cells by limiting their effector function and access to antigen-expressing epidermal cells. In a mouse model, loss or blockade of PD-1 resulted in CD8 + T-cell-mediated epidermal injury. Supporting the relevance of this mechanism in humans, clonally expanded effector CD8 + T cells were identified in skin biopsies from two patients with ICI-associated lichenoid reactions [81].

The inflammatory pathways underlying ICI-associated cutaneous adverse events vary according to the clinical phenotype. In a prospective observational study involving 200 ICI-treated patients, including 139 with cutaneous adverse events, lichenoid and psoriasiform reactions showed increased cutaneous IFN-γ expression, whereas IL-17 A expression was elevated in several inflammatory phenotypes. Eczematous reactions were characterized by increased IL-13 expression, supporting the presence of distinct immunological profiles rather than a uniform inflammatory mechanism [82]. In a separate analysis of three patients with anti-PD-1-associated psoriasis, skin lesions contained infiltrating CD8 + T cells and dendritic cells, together with increased expression of inflammatory mediators including IL-23, IL-17, TNF-α, and IFN-γ [83].

Vitiligo-like depigmentation during anti-PD-1 therapy has been associated with immune responses involving normal melanocytes and melanoma tissue. T-cell receptor sequencing in patients with melanoma identified shared T-cell clonotypes between vitiligo lesions and metastatic tumor samples, suggesting a relationship between antitumor immunity and melanocyte-directed autoimmunity. However, these findings do not establish that identical T-cell clones mediate depigmentation in all affected patients [84].

ICI-associated bullous pemphigoid represents a distinct autoimmune phenotype involving antibodies against BP180 at the dermal–epidermal junction and subsequent inflammatory tissue injury. Although PD-1/PD-L1 blockade is associated with the development of bullous pemphigoid, the precise mechanisms linking checkpoint inhibition to the initiation or amplification of this autoantibody-mediated response remain incompletely understood [85].

Collectively, these findings indicate that ICI-associated cutaneous toxicities involve multiple immune-mediated pathways, including dysregulated cytotoxic T-cell responses, phenotype-specific cytokine activation, and autoantibody-associated inflammation. The mechanistic evidence summarized above derives predominantly from experimental models and adult oncology populations; its applicability to children receiving ICIs for solid tumors has not been established.

#### Clinical Manifestations

Cutaneous adverse reactions associated with targeted therapies display distinct clinical patterns reflecting their molecular mechanisms. Inhibitors of EGFR and MEK commonly cause early-onset acneiform papulopustular eruptions localized to seborrheic areas, often accompanied by xerosis, pruritus, and paronychia. Although papulopustular eruptions may substantially impair quality of life, they rarely necessitate permanent discontinuation of therapy. Interestingly, accumulating clinical evidence indicates that the development of EGFR inhibitor–associated papulopustular rash is associated with improved treatment outcomes, including longer progression-free survival, overall survival, and higher objective response rates, suggesting that rash may serve as a pharmacodynamic marker of effective EGFR inhibition [86]. MEK blockade has additionally been associated with changes in hair texture and lightening of hair color [37, 87]. BRAF inhibitors such as vemurafenib frequently induce photosensitivity, hyperkeratotic plaques, and keratoacanthomas or cutaneous squamous cell carcinomas, although these manifestations are substantially less frequent when BRAF inhibitors are combined with MEK inhibition [88, 89].

mTOR inhibitors typically produce maculopapular or acneiform eruptions, xerosis, stomatitis, and periungual inflammation [48]. PD-1 and PD-L1 immune checkpoint inhibitors are associated with a broad spectrum of immune-mediated skin disorders, including morbilliform, psoriasiform, and lichenoid eruptions, vitiligo-like depigmentation, bullous pemphigoid, and, in rare cases, severe reactions such as Stevens–Johnson syndrome or toxic epidermal necrolysis [85, 90]. Studies of non-treatment-associated vitiligo have demonstrated a role for CXCL10–CXCR3 signaling in the recruitment and function of melanocyte-reactive CD8 + T cells, while IL-15 signaling contributes to the maintenance of skin-resident memory T cells and persistent depigmentation. Although these pathways may also contribute to immune checkpoint inhibitor-associated vitiligo-like depigmentation, their specific involvement in this treatment-related phenotype remains to be established [91, 92].

ALK inhibitors can trigger photosensitive dermatitis or lichenoid eruptions, which occasionally permit successful desensitization and reintroduction of therapy [51, 93]. VEGFR tyrosine kinase inhibitors often cause hand–foot skin reaction characterized by painful palmoplantar hyperkeratosis and fissures, along with xerosis and paronychia [53]. RET inhibitors typically lead to eczematous dermatitis, xerosis, and mucosal dryness or mucositis [57, 94]. NTRK inhibitors have been associated with generally uncommon and nonspecific cutaneous adverse events, including rash and pruritus, although their dermatologic safety profile remains incompletely characterized [62, 95].

BCR-ABL and PDGFR inhibitors are most often associated with maculopapular exanthems, pruritus, and pigmentary alterations, occasionally progressing to lichenoid reactions or Stevens–Johnson syndrome [96]. CDK4/6 inhibitors can provoke photosensitive rashes and dyschromia, with rare vitiligo-like patches [70, 71] HDAC inhibitors cause erythematous, pruritic, or acneiform eruptions [73, 75] whereas dinutuximab beta, a GD2-targeting monoclonal antibody, commonly leads to infusion-related erythema, urticaria, pruritus, and painful inflammatory flares [25, 78].

### Diagnosis

Evaluation of these toxicities combines recognition of characteristic clinical patterns with standardized severity grading and patient-reported outcomes.Comprehensive skin and mucosal examinations are performed and classified according to CTCAE v5.0 criteria, with documentation of lesion distribution (folliculocentric, eczematous, or keratotic patterns), extent of body surface involvement, and symptom intensity measured by pain or pruritus scales. Potential triggers are also identified, including ultraviolet exposure in patients receiving BRAF or ALK inhibitors and mechanical friction in those treated with VEGFR inhibitors associated with hand–foot skin reactions Baseline dermatologic assessment followed by regular surveillance is advisable during targeted therapy. In a retrospective pediatric cohort, dermatologic assessments were performed at baseline and monthly thereafter, reflecting the study-specific monitoring approach rather than an established pediatric surveillance standard [97].

Patient-reported quality-of-life scores such as the CDLQI and serial clinical photographs enhance longitudinal monitoring, while adherence to MASCC and ESMO oncodermatology guidelines improves diagnostic consistency [7, 37].

Skin biopsy is not required to diagnose most targeted therapy–associated eruptions, which are usually recognized clinically from their morphology, distribution, and temporal relationship to the suspected agent. Across drug classes, biopsy should be considered when (i) the morphology is atypical for the suspected agent, or the differential diagnosis includes infection, an unrelated dermatosis, or cutaneous involvement by the underlying malignancy; (ii) the eruption persists or progresses despite appropriate, severity-adapted treatment; (iii) a new, rapidly growing, nodular, or hyperkeratotic lesion raises concern for a cutaneous neoplasm; (iv) blistering, mucosal involvement, or marked skin pain suggests an immune-mediated bullous disorder or a severe cutaneous adverse reaction; or (v) the histopathological diagnosis would directly influence management, including decisions on treatment interruption, permanent discontinuation, or rechallenge [7]. Class-specific indications are outlined below.

Skin biopsy is not routinely required for typical EGFR or MEK inhibitor–associated acneiform eruptions, which are generally diagnosed clinically; however, biopsy may be considered when lesions persist despite appropriate treatment or when the diagnosis remains uncertain [98]. During BRAF inhibitor therapy, new, rapidly growing, hyperkeratotic, or nodular skin lesions warrant prompt dermatologic assessment and histopathological evaluation to exclude keratoacanthoma or cutaneous squamous cell carcinoma [99]. In severe or diagnostically uncertain ALK inhibitor–associated eruptions, skin biopsy may help characterize the inflammatory reaction pattern and distinguish drug-induced dermatitis from alternative diagnoses [100, 101].

The characteristic presentations of VEGFR inhibitor–associated hand–foot skin reaction, mTOR inhibitor–associated stomatitis, and dinutuximab beta–associated infusion-related urticaria and erythema are diagnosed clinically, and biopsy is rarely required unless the presentation is atypical or refractory to treatment. In contrast, lichenoid, persistent, or progressive eruptions during BCR-ABL/PDGFR inhibitor therapy, and new pigmentary changes of uncertain cause, may warrant histopathological evaluation, particularly when the findings would influence the decision to continue, modify, or switch therapy [68, 96].

In patients receiving immune checkpoint inhibitors, blistering or mucosal involvement warrants prompt specialist assessment. When bullous pemphigoid is suspected, diagnostic evaluation should include a lesional skin biopsy for routine histopathology and a perilesional biopsy for direct immunofluorescence, with BP180/BP230 serology as a complementary investigation [85]. Suspected Stevens–Johnson syndrome or toxic epidermal necrolysis requires urgent assessment and management without waiting for histopathological confirmation; skin biopsy may support the diagnosis and help exclude alternative blistering disorders [102, 103]. Bacterial cultures should be obtained when secondary infection is suspected, particularly in purulent or treatment-refractory paronychia [37]. As these biopsy recommendations are largely derived from adult populations, their application in children should be individualized according to the clinical presentation, diagnostic uncertainty, and potential procedural burden. In children, procedural burden can be minimized by restricting biopsy to situations in which the result is likely to change management, by selecting a small punch biopsy from a representative and cosmetically less sensitive site, and, when feasible, by coordinating sampling with other procedures performed under sedation or anesthesia.

Photosensitivity testing, including detailed exposure history or minimal erythema dose determination, is performed mainly for ALK or BRAF inhibitors showing phototoxic features [51]. When clinically relevant oral mucositis or stomatitis is present, standardized mucositis grading may provide a structured approach to documenting severity and treatment response. A practical diagnostic approach begins with identification of the likely causative drug class and clinical pattern, followed by CTCAE severity grading and assessment of aggravating factors such as UV exposure, friction, or secondary infection. Management can then be tailored to the suspected mechanism, with subsequent reassessment guiding dose modification or re-challenge when appropriate. In specific cases, such as adding MEK inhibitors to mitigate paradoxical MAPK activation under BRAF inhibition or gradual desensitization for alectinib hypersensitivity, tailored interventions are recommended [69, 74]. Multidisciplinary evaluation—including dermatology, oncology, and supportive care teams—is essential for persistent grade 2 or higher toxicities, rapidly proliferative or mucosal lesions, and any presentation suggestive of severe immune-mediated reactions such as Stevens–Johnson syndrome or toxic epidermal necrolysis [85].

### Treatment

Management aims to control cutaneous toxicity while avoiding unnecessary interruption of anticancer therapy, with prevention and early intervention playing central roles [7, 37, 104]. Current international guideline recommendations are summarized in Table 2. In the case of EGFR and MEK inhibitors, acneiform eruptions are managed with regular emollient use, broad-spectrum sunscreen, and topical corticosteroids or antibiotics, while oral tetracyclines are added for grade 2 or higher reactions. The role of Cutibacterium acnes in amplifying IL-36γ-driven inflammation may partly explain the benefit of tetracyclines beyond their established anti-inflammatory effects [43]. Emerging evidence also suggests that correction of zinc deficiency may improve EGFR inhibitor–associated xerosis, papulopustular eruptions, paronychia, and eczema in selected patients, although prospective evidence remains limited [105]. Topical vitamin K1 has also been investigated for the treatment of established cetuximab-induced rash, with a randomized clinical trial reporting clinical improvement; however, evidence remains limited, and prophylactic vitamin K1 is not generally recommended by current ESMO guidance [106]. Combined BRAF and MEK inhibition has been consistently associated with a lower incidence of cutaneous squamous cell carcinoma and keratoacanthoma compared with BRAF inhibitor monotherapy [5, 6, 9, 88, 89].

**Table 2:** Comparison of current guideline recommendations for dermatologic toxicities associated with targeted therapies

Organization	Scope of Guidance	Prevention Strategies	Treatment Recommendations*	Pediatric Considerations
MASCC Skin Toxicity Study Group [37]	EGFR inhibitor–associated dermatologic toxicities	Patient education, moisturizers, photoprotection; prophylactic topical corticosteroid plus oral tetracycline for papulopustular eruption	Toxicity-specific management of papulopustular eruption, xerosis/fissures, pruritus, paronychia, mucositis, and other EGFRI-associated toxicities	Primarily adult evidence; no pediatric-specific recommendations
ESMO Clinical Practice Guideline [7]	Dermatologic toxicities associated with anticancer agents	Preventive skin care, photoprotection, and toxicity-specific prophylactic strategies	Severity- and phenotype-based treatment, including topical/systemic therapies and treatment modification when clinically indicated	Predominantly adult evidence; pediatric-specific evidence limited
NCCN [107]	EGFR inhibitor–associated dermatologic and ocular toxicities	Patient education, photoprotection, moisturizers, and prophylactic oral tetracyclines; pre-emptive multiagent skin treatment may reduce rash severity	Toxicity- and severity-based topical or systemic treatment; culture and targeted antimicrobial therapy for suspected infection; temporary treatment interruption for severe toxicity when necessary	Primarily adult evidence; no pediatric-specific recommendations
ASCO [15]	Immune checkpoint inhibitor–related immune-mediated adverse events, including cutaneous toxicities	Patient education and clinical monitoring for early recognition of immune-related cutaneous toxicity	CTCAE- and phenotype-based management of inflammatory, bullous, and severe cutaneous adverse reactions; treatment interruption and systemic immunosuppression according to toxicity severity	Adult guideline; pediatric-specific recommendations not provided

Photosensitivity reactions induced by vemurafenib or ALK inhibitors are managed with strict photoprotection using high-SPF UVA/UVB filters, with short courses of systemic corticosteroids reserved for severe cases [51]. mTOR inhibitor–related rashes and stomatitis are managed with barrier-protective care, topical corticosteroids, antihistamines, and corticosteroid mouth rinses; systemic steroids and temporary dose adjustments are indicated for grade 3 reactions [46, 108, 109]. Immune checkpoint inhibitor–associated cutaneous adverse events require phenotype- and severity-specific management. Mild inflammatory eruptions (grade 1) are generally managed with emollients and topical corticosteroids without treatment interruption. For grade 2 inflammatory dermatitis, temporary interruption of immunotherapy may be considered, together with topical treatment and, when clinically indicated, oral prednisone (0.5–1 mg/kg/day). Grade 3 inflammatory dermatitis generally requires withholding immunotherapy, dermatologic consultation, and systemic prednisone (approximately 1 mg/kg/day), followed by a gradual taper over at least four weeks. Grade 4 reactions require urgent specialist assessment, immediate interruption of immunotherapy, and may necessitate intravenous methylprednisolone (1–2 mg/kg/day). Bullous dermatoses and severe cutaneous adverse reactions, including Stevens–Johnson syndrome and toxic epidermal necrolysis, require separate management pathways and may necessitate permanent treatment discontinuation. These recommendations are primarily derived from adult guidelines and require individualized application in pediatric patients [15].

VEGFR tyrosine kinase inhibitors commonly induce hand–foot skin reaction, which is addressed with keratolytic agents containing urea or salicylic acid, potent topical corticosteroids, analgesic creams, and mechanical off-loading; severe grade 3 HFSR may require temporary treatment interruption or dose reduction in addition to intensive local supportive care [53–55]. Paronychia associated with EGFR or VEGFR inhibition is managed with antiseptic soaks and topical mupirocin, and periungual pyogenic granuloma–like lesions have been reported to respond to topical timolol 0.5% gel [110].

ALK inhibitor–induced lichenoid or photosensitive eruptions generally respond to topical or short systemic corticosteroids, and gradual desensitization has, in selected cases, enabled reintroduction of therapy [93]. Management of RET- and NTRK inhibitor–associated cutaneous adverse events is primarily supportive and may include emollients and topical anti-inflammatory therapy, with oral antihistamines used for symptomatic pruritus [57, 62].

BCR-ABL and PDGFR inhibitor–induced rashes are treated with topical corticosteroids and antihistamines, while short systemic steroid courses are indicated for moderate cases; if lichenoid or severe hypersensitivity reactions recur, switching to another tyrosine kinase inhibitor within the same class is recommended [68, 96]. CDK4/6 inhibitors require strict photoprotection, with supportive management for pigmentary alterations; vitiligo-like depigmentation generally needs observation only [70, 71]. HDAC inhibitor–associated exanthems respond to topical anti-inflammatory agents, while severe eruptions may necessitate a brief course of systemic corticosteroids. Finally, patients receiving dinutuximab beta benefit from antihistamine premedication, close monitoring during infusion, and supportive management of infusion-related erythema, urticaria, and pruritus. Corticosteroids should be reserved for clinically indicated reactions according to the treatment protocol rather than considered routine premedication [25, 78]. Practical prevention and management strategies for targeted therapy–related dermatologic adverse effects are summarized in Table 3.

**Table 3:** Practical considerations for prevention and management of targeted therapy–related dermatologic adverse effects

Recommendation	Benefit-Risk Balance	Resource Requirement
Routine dermatologic monitoring and patient education	Favorable; low anticipated risk	Low
Prophylactic emollients and photoprotection	Favorable; low anticipated risk	Low
Early tetracycline therapy for acneiform eruptions	Potential benefit; age-dependent safety considerations	Low to moderate
Systemic corticosteroids for grade ≥3 immune-mediated toxicities	Potential benefit in selected inflammatory reactions; clinically significant treatment risks	Moderate
Combined BRAF/MEK therapy to reduce proliferative skin lesions	Favorable dermatologic profile compared with BRAF inhibitor monotherapy; regimen-specific adverse effects remain	Moderate
Multidisciplinary dermatology-oncology management	Favorable; additional resource requirements	Moderate
Targeted biopsy and microbiologic testing when clinically indicated	Diagnostic benefit when indicated; procedural burden and risks	Moderate

*Benefit–risk assessments are qualitative judgments based on the available clinical literature and expert guideline recommendations. They do not represent a formal quantitative benefit–risk analysis or a validated evidence-grading system. The strength of supporting evidence varies across interventions, and pediatric-specific data remain limited. Treatment decisions should be individualized according to the therapeutic agent, toxicity phenotype, severity, and patient age

### Prognosis

Many cutaneous toxicities can be controlled without permanent treatment discontinuation when recognized and managed early [7, 104]. Combination BRAF/MEK therapy reduces high-risk proliferative skin lesions versus BRAF monotherapy, improving dermatologic safety while enhancing tumor outcomes [5, 88]. Immune-related dermatoses observed during PD-1/PD-L1 blockade have been reported to correlate with antitumor activity in certain settings (e.g., vitiligo in melanoma) and are manageable with guideline-directed immunosuppression, though rare SJS/TEN carries significant risk and mandates cessation [85, 90]. For VEGFR-TKI HFSR and mTOR-related stomatitis/rashes, structured care pathways and dose adjustment are frequently associated with restoration of function and improvement in quality of life [53, 108].

## Discussion

### Current Guidelines and Practice Alignment

Existing clinical practice guidelines—such as those of the Multinational Association of Supportive Care in Cancer (MASCC), the European Society for Medical Oncology (ESMO), the National Comprehensive Cancer Network (NCCN), and the American Society of Clinical Oncology (ASCO)—offer structured recommendations for the prevention and management of cutaneous toxicities. These recommendations broadly align with the clinical evidence reviewed here, particularly in their emphasis on prevention, severity-based escalation, and early dermatologic involvement. MASCC guidance for EGFR inhibitor–associated dermatologic toxicities emphasizes preventive skin care, patient education, photoprotection, and prophylactic or reactive treatment of papulopustular eruptions, xerosis, pruritus, and paronychia [37]. The more recent ESMO Clinical Practice Guideline extends management recommendations across a broader spectrum of anticancer therapy–related dermatologic toxicities [7]. The NCCN Task Force Report similarly supports proactive management of EGFR inhibitor–associated dermatologic toxicities, including patient education, preventive skin care, photoprotection, and early topical or systemic intervention, while emphasizing that much of the available evidence was historically based on small studies and expert consensus [107]. For immune checkpoint inhibitor–related cutaneous toxicities, the ASCO Clinical Practice Guideline recommends phenotype- and severity-based management, including treatment interruption and systemic immunosuppression for clinically significant immune-mediated reactions when indicated [15].

### Pediatric Adaptation of Adult Dermatologic Management Guidelines

Although established dermatologic toxicity management guidelines provide a useful framework for supportive care during targeted anticancer therapy, their direct application to pediatric patients requires consideration of age, pubertal development, drug-specific toxicity patterns, and the limited availability of pediatric interventional evidence. Pediatric observational studies of MEK inhibitors have identified age-related differences in cutaneous manifestations, with xerotic and eczematous reactions occurring more frequently in younger children and inflammatory acneiform eruptions becoming more prominent in older or postpubertal patients [111–113]. These findings suggest that preventive counseling, clinical surveillance, and treatment selection should be individualized rather than uniformly extrapolated from adult protocols.

The supportive measures described in the Treatment section, including regular emollient use, gentle skin cleansing, photoprotection, and early management of inflammatory or periungual lesions, are applicable to pediatric care but should be adapted to the child’s age, skin condition, and ability to adhere to treatment Pediatric observational studies support baseline dermatologic assessment, caregiver education, and continued surveillance. However, the frequency and structure of dermatologic follow-up vary across clinical settings, and no universally validated surveillance interval has been established for children receiving targeted anticancer therapies. Monitoring should therefore be individualized according to the therapeutic agent, developmental stage, previous cutaneous adverse events, and emerging clinical findings [44, 111, 112].

Pharmacologic management likewise requires pediatric-specific assessment. Topical corticosteroids should be selected according to the affected anatomical site, inflammatory severity, and appropriate potency and duration, while systemic antibiotics should be considered according to clinical indication, age-related safety considerations, and suspected secondary infection rather than prescribed routinely for every acneiform eruption. Pediatric cohorts have documented the use of topical corticosteroids, topical antimicrobials, oral antibiotics, and, in selected refractory cases, oral retinoids; however, these observational findings do not establish comparative treatment efficacy [111, 113]. More recently, expert guidance derived from the ReNeu trial has proposed distinct supportive-care approaches for prepubertal and postpubertal patients receiving mirdametinib, including age-sensitive consideration of systemic tetracyclines and site-specific topical treatments. These recommendations offer a practical framework for MEK inhibitor–associated reactions but have not been validated across all pediatric targeted therapies [11].

Several of the recommendations listed in the Treatment section require specific adaptation in children. Oral tetracyclines, recommended in adult guidelines for grade ≥ 2 EGFR and MEK inhibitor–associated acneiform eruptions, should be selected with attention to age-related safety, including the traditional caution regarding tooth discoloration in children younger than 8 years; the ReNeu-derived guidance accordingly distinguishes prepubertal from postpubertal patients, with greater reliance on topical treatment in younger children [11]. Corticosteroid doses for immune checkpoint inhibitor–associated reactions are expressed per kilogram in adult guidance and can be weight-adjusted in children, but cumulative exposure, effects on growth, and infection risk should be considered whenever prolonged courses are required. Photoprotection during BRAF, ALK, and CDK4/6 inhibitor therapy should rely primarily on protective clothing, shade, and age-appropriate sunscreens, and keratolytic and off-loading measures for hand–foot skin reaction should be adapted to the child’s footwear and daily activities. Adjunctive approaches described in adults, such as zinc supplementation or topical vitamin K1, have not been evaluated in children and should not be used routinely outside clinical studies. By contrast, recommendations for everolimus-associated stomatitis [13] and dinutuximab beta–associated infusion reactions [26, 27] draw substantially on pediatric experience and can be applied more directly, whereas recommendations for ALK, NTRK, RET, CDK4/6, and HDAC inhibitors remain largely extrapolated from adult cohorts and case reports.

Finally, decisions regarding treatment interruption, dose reduction, or rechallenge should be coordinated with the treating pediatric oncologist, taking into account the severity and persistence of dermatologic toxicity, the underlying malignancy, and the potential consequences of interrupting anticancer treatment. Close collaboration between pediatric oncology and dermatology is particularly important for severe, atypical, recurrent, or treatment-refractory eruptions. Overall, the available evidence supports individualized, developmentally appropriate adaptation of adult management principles while highlighting the need for prospective pediatric studies and validated, drug-specific dermatologic care pathways [11, 44, 113]. Evidence regarding dermatologic toxicities associated with emerging pediatric targeted therapies, including FGFR, SHP2, KRAS G12C, and epigenetic inhibitors, remains limited and represents an important area for future investigation.

### Quality and Limitations of the Evidence

The evidence base is heterogeneous in study design, patient population, and reporting of dermatologic outcomes. Only a minority of studies employed prospective designs or standardized dermatologic grading. Most pediatric data derive from mixed-age clinical trials, mechanistic translational studies, or case series extrapolated from adult experience. Controlled pediatric trials focusing specifically on dermatologic outcomes are scarce, which limits the generalizability of the evidence. Reporting bias is evident—cutaneous adverse events are often underreported or aggregated into broad toxicity categories. The use of different grading systems (CTCAE v4 vs. v5, WHO, or institution-specific scales) further complicates meta-analytic synthesis. Nevertheless, similar mechanistic patterns across independent experimental and clinical studies support the biological plausibility of several observed associations. Because this review was designed as a focused narrative synthesis of clinically and mechanistically relevant literature rather than a formal systematic review or meta-analysis, quantitative pooling of outcomes was not performed.

### Controversies and Research Gaps

The contribution of the skin microbiome, particularly C. acnes and Staphylococcus epidermidis, to acneiform eruptions remains an important research question, including whether microbiome-directed interventions have therapeutic value. Genetic susceptibility factors, such as polymorphisms in MAPK pathway regulators or cytokine genes, may predispose certain patients to severe reactions, although these associations remain to be defined. Longitudinal studies are needed to determine whether chronic toxicities such as pigmentary alterations or nail dystrophies have long-term developmental implications in children. Because much of the mechanistic evidence derives from adult or experimental skin models, its applicability to developing pediatric skin requires direct investigation.

The optimal management of immune-related skin toxicity remains an area of active debate. While early corticosteroid use prevents escalation, prolonged immunosuppression may theoretically attenuate antitumor immunity. The balance between efficacy and tolerability therefore requires precise timing and dosage adjustments supported by biomarker-guided monitoring. Similarly, for VEGFR-associated hand–foot reactions, consensus is lacking regarding the prophylactic use of topical keratolytics or systemic anti-inflammatories, and controlled pediatric trials are absent.

An additional area of emerging interest concerns the potential immunomodulatory effects of H1 antihistamines. Although these agents are widely used for symptomatic management of pruritus and urticaria associated with targeted therapies, recent observational studies and meta-analyses have suggested a possible association between concomitant H1 antihistamine use and improved outcomes in patients receiving immune checkpoint inhibitors. The underlying mechanisms remain incompletely understood and may involve modulation of histamine-dependent immune pathways. However, current evidence is insufficient to support the routine use of H1 antihistamines as anticancer agents outside clinical trials, and prospective studies are required to clarify their therapeutic relevance [114, 115].

### Future Directions

Prospective pediatric studies should incorporate standardized dermatologic endpoints, consistent toxicity grading, and patient-reported outcomes. Mechanistic biomarker studies—such as transcriptomic or proteomic profiling of affected skin—could clarify pathway-specific immune signatures and predict toxicity risk before treatment initiation. International guidelines should also address age-specific prevention and management more explicitly Prospective work should therefore involve oncology, dermatology, nursing, and psychosocial care from study design through long-term follow-up. Evidence regarding dermatologic toxicities associated with emerging pediatric targeted therapies, including FGFR, SHP2, KRAS G12C, and epigenetic inhibitors, remains limited and represents an important area for future investigation.

### Clinical Relevance

The available evidence links several recurring patterns of skin toxicity to disruption of pathways that are also required for normal epidermal and immune function. Across drug classes, toxicity most often reflects on-target or paradoxical disruption of MAPK/ERK, PI3K/AKT/mTOR, and JAK/STAT signaling, or immune dysregulation during checkpoint blockade, although the underlying mechanisms and clinical manifestations differ between agents. Understanding these shared and drug-specific mechanisms provides a biological framework for interpreting cutaneous reactions; however, mechanistic similarities do not necessarily imply that management strategies are interchangeable across drug classes. Although these reactions are typically not life-threatening, they can substantially affect treatment adherence, psychosocial well-being, and overall quality of life, and the available clinical evidence suggests that early, mechanism-informed management can reduce toxicity severity and may decrease the need for treatment interruption.

In clinical practice, the assessment of dermatologic adverse events should consider the morphology and severity of the reaction, the suspected causative agent, and the patient’s overall clinical condition. These factors may help distinguish reactions amenable to supportive care from those requiring specialist evaluation or consideration of anticancer treatment modification. Pediatric observational studies have reported age-related differences in cutaneous manifestations during MEK inhibitor therapy, supporting the importance of considering developmental stage when evaluating affected children [111–113].

The clinical application of mechanistic insights should therefore be guided by the available drug-specific evidence and individualized patient assessment. Further prospective pediatric studies are needed to evaluate preventive and therapeutic strategies and to inform the development of standardized dermatologic management pathways for children receiving targeted anticancer therapies.

## Conclusions

Dermatologic toxicities associated with targeted therapies in pediatric oncology reflect disruption of signaling pathways involved in epidermal and immune homeostasis. Understanding these mechanisms may support the development of mechanism-informed prevention and early intervention strategies, potentially reducing treatment-related morbidity and the need for treatment interruptions. However, prospective pediatric studies are needed to establish the effectiveness of these approaches and their impact on quality of life. Dermatologic assessment and patient education should therefore form part of supportive care for children receiving targeted anticancer therapy.

## Data Availability

No datasets were generated or analysed during the current study.

## Key References

- Needle CD, et al. Cutaneous Toxicities of MEK Inhibitor Use in Children: A Comparison of Binimetinib and Selumetinib. Pediatr Dermatol. 2025;42:67–72.
  - This pediatric study provides detailed characterization of MEK inhibitor–associated cutaneous toxicities and directly compares binimetinib with selumetinib. It identifies age- and agent-specific toxicity patterns and demonstrates the clinically relevant impact of dermatologic adverse events on treatment modification.
- Friedland R, et al. Cutaneous Reactions in Pediatric Patients Treated with MEK Inhibitors: A Retrospective Single-Center Study. Dermatology. 2024;240:565–571.
  - This pediatric cohort study characterizes the frequency, clinical spectrum, and severity of MEK inhibitor–associated cutaneous adverse events. The findings highlight that pediatric toxicity patterns may differ from those observed in adults and underscore the role of dermatologic care during prolonged targeted therapy.
- Azizi AA, et al. Consensus recommendations on management of selumetinib-associated adverse events in pediatric patients with neurofibromatosis type 1 and plexiform neurofibromas. Neurooncol Pract. 2024;11:515–531.
  - This multidisciplinary consensus provides pediatric-specific recommendations for the prevention, monitoring, and management of selumetinib-associated adverse events in patients with NF1-associated plexiform neurofibromas. It translates available clinical evidence and expert experience into practical strategies for maintaining treatment while minimizing toxicity.
- Morelli M, Carbone ML, Scaglione GL, et al. Identification of immunological patterns characterizing immune-related psoriasis reactions in oncological patients in therapy with anti-PD-1 checkpoint inhibitors. Front Immunol. 2024;15:1346687.
  - This mechanistic study demonstrates that anti-PD-1–induced psoriasis is characterized predominantly by adaptive immune activation, with prominent CD8+ T-cell and dendritic-cell infiltration and increased expression of psoriasis-associated inflammatory cytokines. These findings provide direct human tissue evidence for the immune mechanisms underlying checkpoint inhibitor–associated psoriasiform reactions.
- Moertel CL, Hirbe AC, Shuhaiber HH, et al. ReNeu: A Pivotal, Phase IIb Trial of Mirdametinib in Adults and Children With Symptomatic Neurofibromatosis Type 1–Associated Plexiform Neurofibroma. J Clin Oncol. 2025;43:716–729.
  - This pivotal phase IIb study established the clinical activity and safety of mirdametinib in adults and children with symptomatic NF1-associated plexiform neurofibromas. The trial provides contemporary evidence supporting MEK inhibition as an expanding therapeutic strategy in pediatric NF1 and highlights the need for recognition and management of treatment-associated adverse events.
